# An Effective Antifreeze Protein Predictor with Ensemble Classifiers and Comprehensive Sequence Descriptors

**DOI:** 10.3390/ijms160921191

**Published:** 2015-09-07

**Authors:** Runtao Yang, Chengjin Zhang, Rui Gao, Lina Zhang

**Affiliations:** 1 School of Control Science and Engineering, Shandong University, Jinan 250061, China; E-Mails: runtao-sd@163.com (R.Y.); gaorui@sdu.edu.cn (R.G.); zlnabc2010@163.com (L.Z.); 2 School of Mechanical, Electrical and Information Engineering, Shandong University, Weihai 264209, China

**Keywords:** antifreeze proteins, ensemble method, random forest, majority voting

## Abstract

Antifreeze proteins (AFPs) play a pivotal role in the antifreeze effect of overwintering organisms. They have a wide range of applications in numerous fields, such as improving the production of crops and the quality of frozen foods. Accurate identification of AFPs may provide important clues to decipher the underlying mechanisms of AFPs in ice-binding and to facilitate the selection of the most appropriate AFPs for several applications. Based on an ensemble learning technique, this study proposes an AFP identification system called AFP-Ensemble. In this system, random forest classifiers are trained by different training subsets and then aggregated into a consensus classifier by majority voting. The resulting predictor yields a sensitivity of 0.892, a specificity of 0.940, an accuracy of 0.938 and a balanced accuracy of 0.916 on an independent dataset, which are far better than the results obtained by previous methods. These results reveal that AFP-Ensemble is an effective and promising predictor for large-scale determination of AFPs. The detailed feature analysis in this study may give useful insights into the molecular mechanisms of AFP-ice interactions and provide guidance for the related experimental validation. A web server has been designed to implement the proposed method.

## 1. Introduction

The temperature in cold areas sometimes drops to below −40 ${}^{\circ }$C [1]. To survive at subzero temperatures, many overwintering organisms have developed a high level of freezing tolerance to protect themselves from fatal ice crystal growth [2]. The antifreeze effect is largely due to a family of antifreeze proteins (AFPs) that were first recognized in the Antarctic fishes by DeVries [3] and later identified in a wide range of organisms, including bacteria [4], fungi [5], plants [6] and insects [7]. AFPs have the ability to adsorb onto the surface of ice crystals and inhibit their growth [8], which if left uncontrolled would be fatal to cells. The interaction between AFPs and ice crystals lowers the freezing temperature of ice without significantly affecting the melting temperature, a phenomenon referred to as thermal hysteresis [9]. Excellent progress has been made in the study of antifreeze-ice interactions. Kuiper *et al.* [10] presented a theoretical three-dimensional model of a plant antifreeze protein from Lolium perenne, which can be conducive to deciphering the underlying mechanisms of the properties of antifreeze proteins. Guz *et al.* [11] revealed the functional annotation of a putative antifreeze protein gene. However, as the details of the antifreeze effect are difficult to test experimentally, the ice-binding mechanisms of antifreeze proteins are not completely understood [12]. Some emphasized that hydrogen bonding to ice water molecules was the major driving force of the AFP-ice association [13,14]. Some suggested that hydrophobic interactions could be the main contributor to the AFP-ice association [2,15]. Thus, accurate identification of AFPs may provide important clues to decipher the underlying mechanisms of AFPs in ice-binding. Ultimately, knowledge about the ice-binding mechanisms of antifreeze proteins may allow the design of an improved or more efficient macromolecular antifreeze.

AFPs have a wide range of applications in numerous fields due to the role of their antifreeze property in the protection of tissue or cell damage by freezing [16]. The presence of AFPs may improve the quality of frozen foods by inhibiting recrystallization and maintaining a smooth texture [16,17]. There is rising evidence that AFPs have potential applications in agriculture for improving the production of crops and fishes in cooler climates [18]. AFPs are also used to preserve cells, tissues and organs for transplant or transfusion in medicine at a low temperature [19]. The other proposed applications of AFPs are found in cryosurgery of tumors and therapy for hypothermia [20]. However, as indicated in [21], the quantity of AFPs that may produce superior performance at the molecular level is insufficient for practical use. The identification of AFPs may facilitate the selection of the most appropriate AFPs for several industrial and biomedical applications.

The AFPs show great diversity in their primary sequences and structures [8]. Distinguishing an antifreeze protein from a non-antifreeze protein has challenged the antifreeze field for some considerable time [22]. With the avalanche of genome sequences generated in the postgenomic age, various computational methods based on sequence information have been developed for identification of AFPs. Kandaswamy *et al.* [23] proposed the first computational program called AFP-Pred for the prediction of antifreeze proteins from protein sequences. Zhao *et al.* [24] developed a different predictor named AFP-PSSM utilizing support vector machine (SVM) and position-specific scoring matrix (PSSM) profiles. Recently, according to Chou’s pseudo amino acid composition-based protein features, Mondal and Pai [25] proposed a predictor called AFP-PseAAC (pseudo amino acid composition) to identify AFPs.

Though these methods have facilitated the identification of AFPs to some extent, some limitations should be noted. First, earlier work did not give a real solution to the class imbalance problem. The existing methods for predicting AFPs [23,24,25] have tried to change the distribution of positive and negative samples by randomly selecting AFPs and non-AFPs with the same size as the training set. However, they failed to make full use of the negative sample information in the original dataset. Second, the existing methods did not take the protein sequence features directly related to the binding properties of AFPs into consideration, such as the disorder score, solvent accessible surface and functional domains. Third, the methods of feature extraction in most of the papers were based on a single technique. Multiple feature types have not been investigated simultaneously to get a more robust and discerning feature set. It is inevitable that some useful information would be missed. Therefore, further development for identifying AFPs is definitely needed for the above-mentioned limitations.

**Figure 1:**
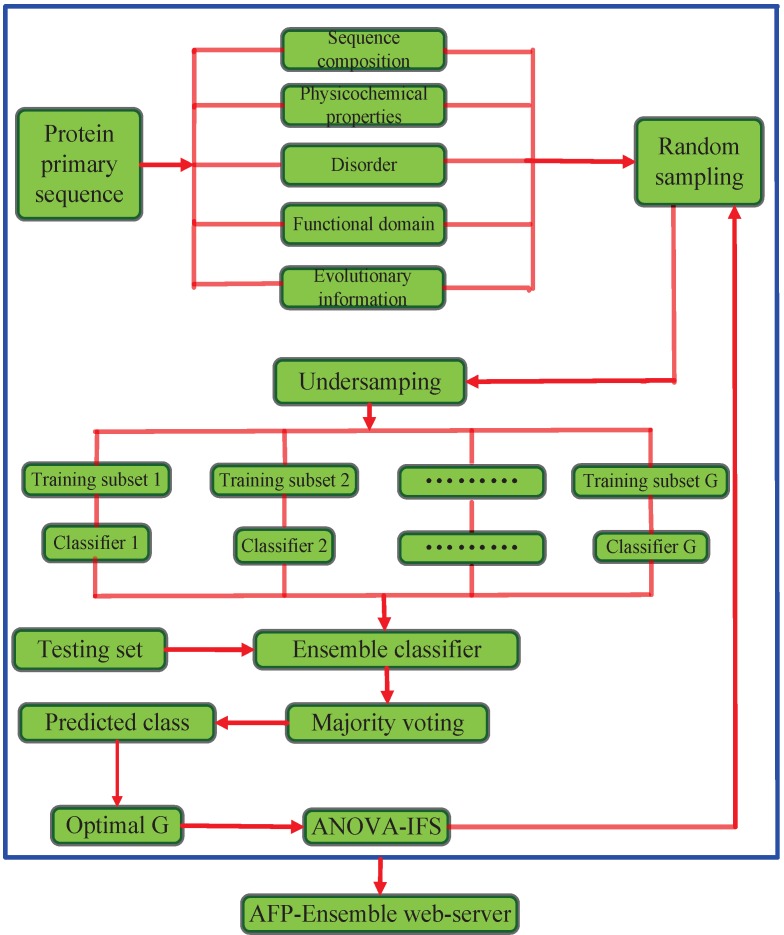
The system architecture of the proposed method. ANOVA-IFS: Analysis of variance with incremental feature selection.

In this paper, we propose a novel AFP classification system (AFP-Ensemble) that performs ensemble classification of samples based on discriminatory capabilities of hybrid feature spaces. The proposed method is implemented in the following steps. (i) Protein sequences are mapped into feature vectors. The feature space is constructed from different types of sequence-derived features, *i.e.*, sequence composition, physicochemical properties, disorder, functional domain and evolutionary information; (ii) Negative samples in the training set are randomly sampled to make sure that the sampled negative samples are *G* times the number of the positive samples in the training set, where G={3,5,7,9,11,13,15}; (iii) The training set is divided into *G* training subsets through the undersampling approach; (iv) The *G* training subsets respectively train random forest classifiers to form ensemble classifiers; (v) Based on the ensemble classifiers, the predicted class labels of the test set are determined through the majority voting method. The parameter *G* is determined based on the prediction performance of the full feature space; (vi) With the optimal parameter *G*, the analysis of variance with incremental feature selection (ANOVA-IFS) procedure is employed to select high discriminative features from the hybrid feature space. To be easy to access and utilize by the public, the presented approach is realized on a user-friendly web-server called AFP-Ensemble. The system architecture of the proposed method is illustrated in Figure 1.

## 2. Results and Discussions

### 2.1. The Impact of the G Value on the Prediction Performance

In order to reduce computational complexity, random sampling is adopted in this study to sample negative samples. As mentioned in Section 2.4, parameter *G* (G={3,5,7,9,11,13,15}) is defined as the ratio of the number of negative samples to positive ones after random sampling, which may have a significant impact on the prediction performance.

**Figure 2:**
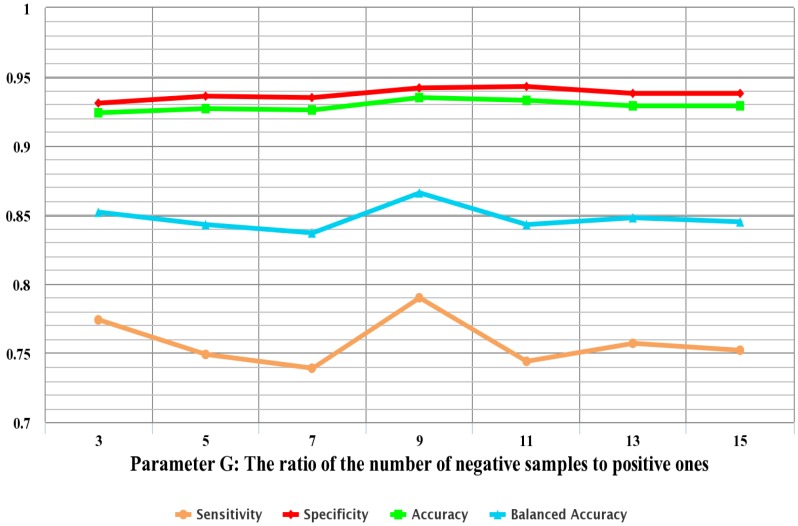
The 10-fold cross-validation performance trained with different values of *G*. Parameter *G* is defined as the ratio of the number of negative samples to positive ones after random sampling.

For different values of *G*, ensemble classifier models based on the full feature space are built and 10-fold cross-validations are carried out. Figure 2 displays the performance of each model with different values of *G*. The results show that *G* has a certain impact on prediction performance. As shown in Figure 2, the overall performance first increases continuously to a maximum value and then drops slightly with the increase of *G*. This phenomenon may be due to the fact that much redundant information is contained with large *G*, while with small *G*, much useful information is lost. The sensitivity, accuracy and balanced accuracy reach maximums with G=9. Thus, nine is selected as the best ratio of the number of negative samples to positive ones after random sampling in the following implementation.

### 2.2. Feature Selection Results

By running the ANOVA algorithm as given in Section 2.5, the ranked feature list (see Table S1) is obtained on the basis of each feature’s relevance to the classes of samples. Within the list, a feature with a smaller index indicates that it is a more important feature for AFP prediction. Such a list of ranked features will be used for searching the optimal feature set in the following IFS procedure.

**Figure 3:**
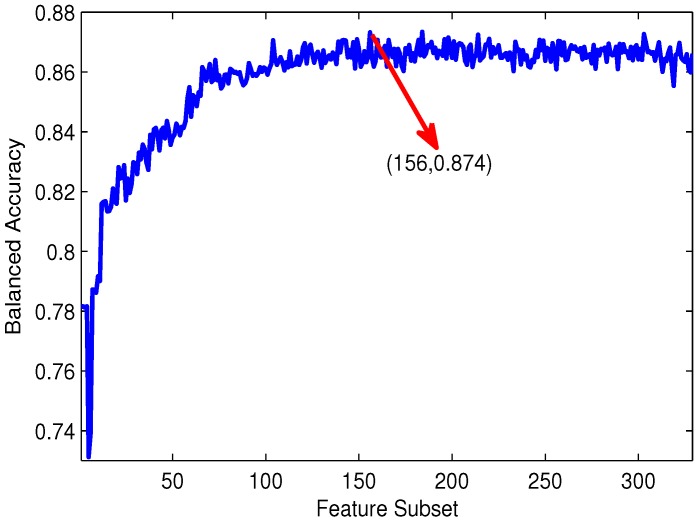
The IFS curve: the values of balanced accuracy against feature subsets. The maximum value of balanced accuracy is 0.874 when the top 156 features are selected. These 156 features are considered as the optimal feature set of our method.

By adding features one by one from the top of the feature list to the bottom, 329 different feature subsets are obtained. The individual predictor is then accordingly built for each feature subset and evaluated by 10-fold cross-validation. The IFS curve is plotted in Figure 3, which reveals the relation between the balanced accuracy and each feature subset.

In the initial phase, the features are too few to contain enough information for antifreeze protein prediction. As shown in Figure 3, the feature selection curve almost monotonically increases in the initial phase. Afterwards, this curve is basically stable with the increase of the number of features. In addition, several oscillations occur in the whole curve. This phenomenon is due to the fact that not all features contribute to good prediction performance. Redundant information may be introduced with a new feature added. The rank of features based on the analysis of variance and the correlation between features has a direct effect on the selection of the maximum. The features containing more useful information, but less redundant information corresponding to maximum balanced accuracy are regarded as the optimal features. The peak of the IFS curve appears with balanced accuracy of 0.874 when the first 156 features are selected. These 156 features are deemed to form the optimal feature set to identify AFPs.

To evaluate our feature selection method, the prediction performance on the original feature set has been measured and then compared to that on the optimal feature set. As can be seen from Table 1, the performance using the optimal feature set is superior to that of the original feature set, with the results of sensitivity, specificity, accuracy and balanced accuracy increasing from 0.790, 0.942, 0.935 and 0.866 to 0.801, 0.946, 0.939 and 0.874, respectively. The specificity of the optimal feature set is also comparable to that of the original feature set. These results demonstrate that the original feature set really contains redundant information or noise. The ANOVA-IFS method makes a certain contribution to picking out informative features.

**Table 1:** Prediction results of the original feature set and the optimal feature set.

Feature Set	No. of Features	Sensitivity	Specificity	Accuracy	Balanced Accuracy
Original feature set	329	0.790	0.942	0.935	0.866
Optimal feature set	156	0.801	0.946	0.939	0.874

### 2.3. Analysis of the Feature Contribution

**Figure 4:**
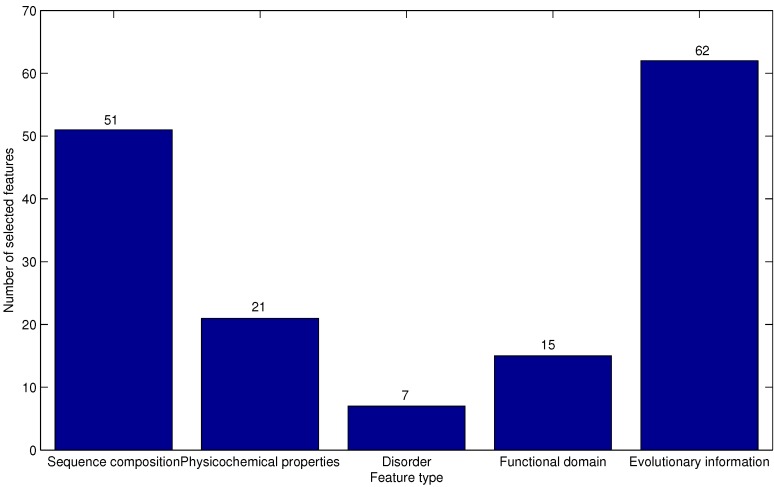
Distribution of each type of feature in the optimal feature set. The five types of features are derived from sequence composition, physicochemical properties, disorder, functional domain and evolutionary information, respectively.

As described in Section 2.2, there are five types of features derived from sequence composition, physicochemical properties, disorder, functional domain and evolutionary information. To discover the different contributions of various types of features, the distribution of each type of feature in the optimal feature set is investigated and depicted in Figure 4.

The majority (62/156 = 0.397) of the selected features are extracted from evolution information, which provides further evidence that evolution information plays important roles in AFP-ice interactions. Actually, evolutionary conservation usually reflects important biological function [26]. Furthermore, the sequence composition-based features account for a second higher proportion of the optimal feature set. This may imply that sequence composition plays an irreplaceable role for the prediction of AFPs. Few features extracted from the functional domain and disorder are within the optimal feature set. This phenomenon may be due to the fact that there are fewer features extracted from functional domain and disorder in the original feature set.

**Figure 5:**
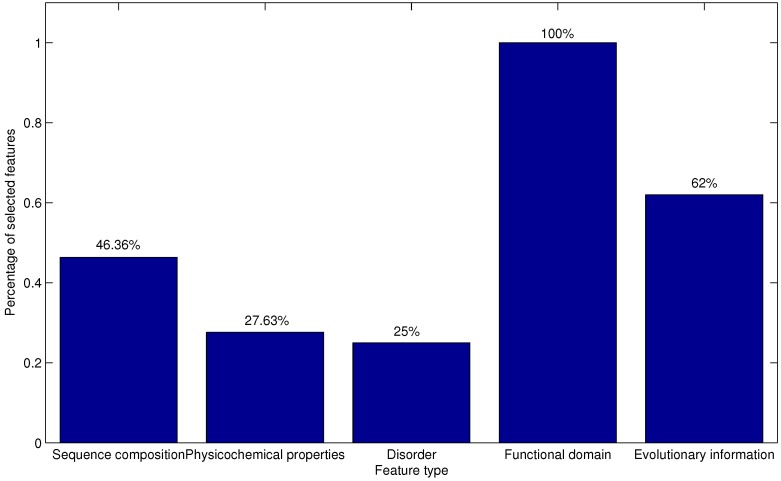
The percentage of the selected features accounting for the corresponding feature type. The five types of features are derived from sequence composition, physicochemical properties, disorder, functional domain and evolutionary information, respectively.

Figure 5 gives the percentage of the selected features accounting for the corresponding feature types. As depicted in Figure 5, the percentages of the five feature types that are chosen to form the optimal feature set are all more than 25%. In addition, it is interesting to note that features from the functional domain are all in the optimal feature set. Most of these functional domains are unconcerned with the antifreeze domain. This result indicates that except antifreeze domains, the other functional domains (Table 2) may have an effect on the antifreeze effect. Features extracted from functional domains in this study are consist with the result of previous work [8] that some underlying, shared structural elements or properties are responsible for the antifreeze effect. These features may give useful insights into the molecular mechanisms of AFP-ice interactions and provide guidance for the related experimental validation. These results indicate that all five types of features contribute to the prediction of AFPs. Different feature extraction strategies dig out diverse types of information from the protein sequences. No single type of feature could undertake the task of AFP prediction accurately. The prediction model integrates multiple sources of descriptors for protein sequences in an attempt to enhance prediction performance. These features may provide important information for identifying the potential AFPs.

### 2.4. Imbalanced Learning Effects

**Figure 6:**
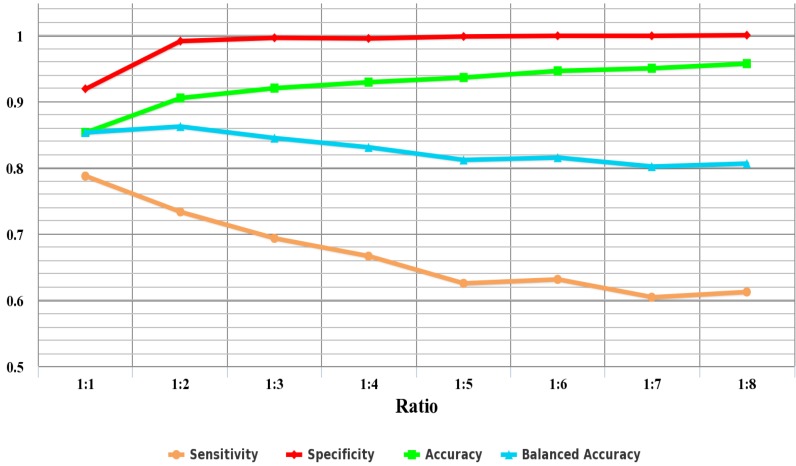
The performance with different ratios between positive and negative samples in the training set without random sampling. The ratios of the number of positive samples to negative ones are from 1:1 to 1:8, respectively.

One challenge in training classifiers comes from the fact that the available dataset is highly unbalanced. The number of AFPs is relatively small compared to that of non-AFPs. To analyze the impact of the scale of negative dataset on prediction performance, negative datasets of *N*, 2N,…, and 8N are randomly extracted from the training dataset, where *N* is the scale of the positive dataset. The eight negative datasets plus the positive dataset from the training dataset then constitute eight new training datasets with the ratios of the number of positive samples to negative ones from 1:1 to 1:8. The performance of prediction systems trained with different ratios is shown in Figure 6 and listed in Table 2.

**Table 2:** The performance with different ratios between positive and negative samples in the training set without random sampling. The ratios of the number of positive samples to negative ones are from 1:1 to 1:8, respectively.

Ratio	Sensitivity	Specificity	Accuracy	Balanced Accuracy
1:1	0.787	0.919	0.853	0.853
1:2	0.733	0.991	0.905	0.862
1:3	0.693	0.996	0.920	0.845
1:4	0.666	0.995	0.929	0.831
1:5	0.625	0.998	0.936	0.812
1:6	0.631	0.999	0.946	0.815
1:7	0.604	0.999	0.950	0.802
1:8	0.612	1.000	0.957	0.806

As shown in Figure 6, the specificity is gradually improved with the increase of negative samples. On the contrary, there is a declining trend for the sensitivity. At a ratio of 1:8, the specificity even reaches one, while the sensitivity dramatically decreases to 0.612. This phenomenon demonstrates that the imbalanced problem will lead to most of the samples being classified as the majority class. These results also indicate that the prediction performance is significantly affected by the imbalanced training dataset. In addition, the accuracy increases with the increase of the number of negative samples, from 0.853 to 0.957, which proves that the accuracy is not a good measure for the imbalanced problem. However, balanced accuracy tends to drop accompanied by higher ratios that indicate more imbalanced datasets. Therefore, for the imbalanced training dataset, it is reasonable that balanced accuracy is chosen as the performance measure to select the optimal features.

### 2.5. Ensemble Learning Results

Based on the results of individual RF-modules, the ensemble learning method attempts to combine different models into a consensus classifier by majority voting. To evaluate the effectiveness of our ensemble method to overcome the imbalanced problem, Table 3 shows the prediction results with or without the ensemble method. After directly performing the 10-fold cross-validation on the training dataset without sampling, the accuracy and specificity achieved are as high as 0.979 and 0.999, respectively. However, the sensitivity is as low as 0.569 due to the imbalanced data size. However, with the ensemble method, the prediction performance achieves a more balanced sensitivity (0.801) and specificity (0.946). The value of balanced accuracy reaches 0.874, far better than that of the unsampled dataset. These results reveal that the ensemble method can effectively solve the imbalanced problem in the antifreeze protein training dataset.

**Table 3:** Prediction results with or without the ensemble method.

Method	Sensitivity	Specificity	Accuracy	Balanced Accuracy
Without ensemble	0.569	0.999	0.979	0.784
With ensemble	0.801	0.946	0.939	0.874

### 2.6. Performance Affected by Dataset Size

The core of statistical learning algorithms is learning prediction rules from training samples. To study the prediction performance affected by the dataset scales, 10%, 20%, …, 80% of the whole positive dataset and 10%, 20%, …, 80% of the whole negative dataset are randomly chosen to construct the training dataset, respectively. The performance of prediction systems trained with the eight training datasets is listed in Table 4. With the increase of the training dataset size, the specificity and accuracy have no obvious changes, while the sensitivity and balanced accuracy are improved significantly. These results reveal that it is important to collect as many training samples as possible to make the learning rules more accurate. This is particularly important when studying the small sample problems where experimentally-derived knowledge is very limited. However, previous methods [23,24,25] only randomly take 300 non-AFPs from 9493 non-AFPs as the training dataset, which may adversely affect the prediction performance. The training dataset in this study is composed of 80% of the whole positive dataset and 80% of the whole negative dataset in an attempt to enhance the prediction performance.

**Table 4:** Prediction performance with different training dataset sizes. The training dataset is composed of 10%, 20%, …, 80% of the whole positive dataset and 10%, 20%, …, 80% of the whole negative dataset, respectively.

Training Dataset Size	Sensitivity	Specificity	Accuracy	Balanced Accuracy
10%	0.326	0.971	0.940	0.649
20%	0.360	0.966	0.937	0.663
30%	0.422	0.962	0.936	0.692
40%	0.508	0.954	0.932	0.731
50%	0.560	0.957	0.938	0.759
60%	0.640	0.953	0.938	0.800
70%	0.719	0.949	0.937	0.834
80%	0.801	0.946	0.939	0.874

### 2.7. Comparison with the Existing Methods on the Independent Testing Dataset

To evaluate the prediction performance of the current method objectively, comparisons are carried out for AFP-Ensemble and previously-published methods on the independent testing dataset. Table 5 reports the detailed prediction results obtained by AFP-Pred [23], AFP-PSSM [24], AFP-PseAAC [25] and AFP-Ensemble. AFP-Pred combines the predicted secondary structure information and physicochemical properties. AFP-PSSM is mainly based on the information extracted from evolutionary profiles. AFP-PseAAC explores the effect of sequence order information in the prediction of AFPs by using Chou’s pseudo amino acid composition-based features.

**Table 5:** The prediction results compared to those of other methods on the independent testing dataset. Pred, prediction; PSSM, position-specific scoring matrix; PseAAC, pseudo amino acid composition.

Reference	Method	Sensitivity	Specificity	Accuracy	Balanced Accuracy
[23]	AFP-Pred	0.847	0.840	0.843	0.844
[24]	AFP-PSSM	0.759	0.933	0.930	0.846
[25]	AFP-PseAAC	0.862	0.847	0.848	0.855
This study	AFP-Ensemble	0.892	0.940	0.938	0.916

As shown in Table 5, AFP-Ensemble achieves the highest sensitivity of 0.892, specificity of 0.940, accuracy of 0.938 and balanced accuracy of 0.916. Specifically, the balanced accuracy of AFP-Ensemble is 0.072, 0.070 and 0.061 higher than that obtained by AFP-Pred, AFP-PSSM and AFP-PseAAC, respectively. The specificity and accuracy of AFP-Ensemble are far better than those of AFP-Pred and AFP-PseAAC. Although the specificity and accuracy of AFP-PSSM are comparable to our method, the sensitivity is much lower (0.759) compared to AFP-Ensemble. By comparing to the other three competing methods, it is worth pointing out that AFP-Ensemble has a fairly good capability to predict AFPs.

In addition, we randomly select a subset from the original training dataset as an independent dataset. The select procedure is iterated 10 times. Then, the proposed method is evaluated on the 10 independent datasets. Prediction results show that the performance on the 10 independent datasets is similar, which indicates that the proposed method is reliable and robust.

The outstanding performance of the current method may be attributed to three aspects. (i) The training dataset makes full use of the negative sample information in the original training dataset; (ii) The hybrid features, extracted from some inherent properties related to AFP-ice interactions, take full advantage of the supplementary information from different feature types to improve classification performance and prediction robustness; (iii) The imbalanced problem is effectively addressed by a random forest-based ensemble method.

### 2.8. AFP-Ensemble Web Server

In order to facilitate easy use of our AFP prediction model by the public, an AFP-Ensemble web server is established to identify AFPs. The AFP-Ensemble web server provides a user-friendly interface and predictive results. Neither registration nor license acquisition is required for academic usage of this server. Users can paste protein sequences with a FASTA format in the text box area or input the UniProtKB ID of the query protein for prediction. After protein sequences are submitted to our server, the user will be redirected to the result page that includes input information and predicted result. If an email address is given to the server during the task submission, a hyperlink to the result page will be sent to the user’s E-mail once the task is accomplished. The dataset used in our study is also provided at the website. The web server is available at [27].

## 3. Experimental Section

### 3.1. Datasets

The dataset composed of 481 AFPs and 9193 non-AFPs, downloaded from [28], is employed to construct the training dataset and independent testing dataset. Two hundred twenty one AFPs are taken from seed proteins of the Pfamdatabase. To enrich the dataset, a PSI-BLAST (position-specific iterated basic local alignment search tool) search for each sequence against the non-redundant sequence database is performed. None of the proteins has 25% sequence identity to any other in the dataset. The final positive dataset contained 481 non-redundant antifreeze proteins. The negative dataset was constructed from 9193 seed proteins (representative members) of Pfam protein families, which are unrelated to antifreeze proteins. These positive and negative samples are employed to construct the training dataset and independent testing dataset.

To obtain high quality data, protein sequences with less than 50 amino acids are screened out, because their information is redundant and not integral. Protein sequences deleted from UniProt or containing nonstandard amino acids, such as “B”,“J”, “O”, “U”, “X” and “Z”, are also removed because their meanings are ambiguous. Thus, the final dataset consists of 464 AFPs and 9083 non-AFPs.

The dataset used in this paper for performance analysis and comparison is divided into two parts: the training dataset and independent testing dataset. In order to not change the distribution of protein samples in the dataset, 20% of the positive dataset and 20% of the negative dataset are randomly chosen to construct the independent testing dataset. The remaining protein sequences are utilized as the training dataset. The number of samples in each dataset is given in Table 6. The final training and independent testing datasets are available in Table S1.

**Table 6:** The number of samples in the training dataset and independent testing dataset.

Dataset	AFPs	Non-AFPs	Total
Training dataset	371	7266	7637
Independent testing dataset	93	1817	1910

### 3.2. Feature Extraction

To develop a powerful predictor, it is significant to formulate protein samples with a comprehensive and proper feature vector that could really reflect the intrinsic correlation with the desired target [29]. To realize this, some sequence-derived encoding schemes that have been observed to be closely related to the AFPs are employed to represent each protein sequence.

Previous research work indicated that an individual feature extraction strategy can only represent a partial target’s knowledge. Feature extraction methods from different sources can complement each other in capturing the valuable information of protein samples [30,31]. Hybrid features extracted from sequence composition, physicochemical properties, disorder, the functional domain and evolutionary information are employed in this study for the numerical description of protein samples. In the following subsections, these feature extraction strategies will be explained in detail.

#### 3.2.1. Sequence Composition

It has been reported that functional groups positioned to match the ice lattice on a particular plane may lead to ice binding and antifreeze activity [32]. We categorize amino acids into 10 functional groups based on the presence of side chain chemical groups, such as phenyl (F/W/Y), carboxyl (D/E), imidazole (H), primary amine (K), guanidino (R), thiol (C), sulfur (M), amido (Q/N), hydroxyl (S/T) and non-polar (A/G/I/L/V/P) [33]. Based on 10 functional groups, amino acid composition (AAC) and dipeptide composition (DPC) are computed for every sequence.

The amino acid composition (AAC) is calculated using the following formula:

(1) $$f\left(g_{i}\right)=\frac{N\left(g_{i}\right)}{L},\left(i=1,2,\cdots ,10\right)$$

where $N(g_{i})$ denotes the number of functional group $g_{i}$ in a given protein sequence and *L* is the sequence length of the given protein sample.

The dipeptide composition (DPC) is defined as:

(2) $$f\left(g_{i}g_{j}\right)=\frac{N\left(g_{i}g_{j}\right)}{L-1},\left(i,j=1,2,\cdots ,10\right)$$

where $N\left(g_{i}g_{j}\right)$ denotes the number of the dipeptides encoded as “$g_{i}g_{j}$” in a given protein sequence.

#### 3.2.2. Physicochemical Properties

A physicochemical property is the most intuitive feature for protein biochemical reactions [34]. The specificity and diversity of a protein’s structure and function are largely attributed to various physicochemical properties of amino acids. Incorporating features extracted from physicochemical properties might contribute to a prospective improvement for protein attribute predictions. A previous study has shown that the feature groups used to reveal global and local discriminatory information can both effectively enhance the prediction performance [35]. The global and local features extracted from physicochemical properties will be explained in detail.

Previous studies have chosen several antifreeze proteins to investigate their physicochemical properties [13,17,18]. The results suggest that the physicochemical parameters, including theoretical isoelectric point (pI), total number of negatively-charged residues of Asp and Glu, total number of positively-charged residues of Arg and Lys, the instability index, the aliphatic index and the grand average of hydropathicity (GRAVY), may provide important clues to decipher the mechanism of AFP binding. In this study, these 6 physicochemical parameters are computed using Expasy’s ProtParam [36] and selected as global features extracted from physicochemical properties.

The pseudo amino acid composition (PseAAC) [37] was proposed to avoid losing local sequence order information hidden in protein sequences and, hence, has rapidly penetrated into almost all of the fields of protein attribute predictions [38,39,40,41]. For a detailed description about its recent development and applications, refer to the comprehensive review [29]. Various modes of PseAAC by extracting different features from protein sequences were proposed in [42,43,44]. In this work, we adopt the auto covariance (AC) model to capture local discriminatory information from physicochemical properties.

Seven physicochemical properties, including hydrophobicity, hydrophilicity, net charge, van der Waals, free energy of solution in water, side chain interaction parameter and average accessible surface area, are taken into account to calculate the AC model on the basis of the following reasons. (i) The hydrophobicity and hydrophilicity of the native amino acids play an important role in protein folding, interior packing, catalytic mechanism, as well as the interaction with other molecules [45]; (ii) Charged amino acids tend to form an internal salt bridge, which is considered to maintain a long helix for stable structure; (iii) As an alternative to the H-bond model, van der Waals and hydrophobic interactions were also suggested to play important roles in AFP binding [2]; (iv) AFPs should obviously be water-soluble and interact with ice [2], which may be attributed to the free energy of the solution in water and the side chain interaction parameter; (v) The ice-binding sites of AFPs are relatively flat and engage a substantial proportion of the protein’s surface area in ice binding [8]. To facilitate contacting ice, the binding residues are always well exposed to solvents. The values of these properties can be obtained from the Amino Acid Index database [46].

To encode a protein sequence, AC variables describe the average interactions between two residues with a certain distance throughout the whole sequence. The AC variables are calculated through the following equation.

(3) $$\mathrm{AC}\left(j,\lambda \right)=\frac{1}{L-\lambda }\sum_{i=1}^{L-\lambda }\left(P_{ij}-\frac{1}{L}\sum_{i=1}^{L}P_{ij}\right)\left(P_{\left(i+\lambda \right)j}-\frac{1}{L}\sum_{i=1}^{L}P_{ij}\right)\;$$

where *j* represents one physicochemical property, *L* is the length of the protein sequence, $P_{ij}$ is the *j*-th physicochemical property value of the amino acid at the *i*-th position in the sequence and λ is the distance between one residue and its neighbor at a certain number of residues away. Thus, AC encodes a protein sequence with the seven physicochemical properties into a vector of size $7\times \lambda_{max}$, where $\lambda_{max}$ is the maximum of λ ($\lambda =1,2,\cdots ,\lambda_{max}$) and chosen as 10.

#### 3.2.3. Disorder

A protein region is defined as “disorder” if it fails to form well-defined three-dimensional structures in its native state [47]. The disorder regions are always rich in binding sites and carry out important roles in regulating protein functions, including enzyme catalysis, cell signaling pathways and ligand binding [48]. One can think of AFPs binding to ice as a receptor-ligand interaction (in which the AFP is the receptor and ice is the ligand) [22], which suggests that there may exist a certain relationship between AFP-ice binding and disorder regions.

The disorder predictor “VSL2” [49] is employed in this study to calculate the disorder score of each residue in a given protein sequence. The disorder score ranges from 0 to 1, where the higher the score is, the more likely the residue lacks a fixed structure. The following 28 features are designed to encode each protein sequence: (i) mean/standard deviation of all residues’ disorder scores (2 features); (ii) number of disorder/non-disorder segments (2 features); (iii) minimum/maximum length of disorder/non-disorder segments (4 features); and (iv) the average disorder score of each native amino acid (20 features).

#### 3.2.4. Functional Domain

It is widely accepted that the protein structure could directly reveal its function mechanics, and thus, the availability of structure information about a given protein should be conducive to improving the performance of protein attribute predictions [50]. Protein domains are distinct functional and/or structural units in transcriptional activities and other intermolecular interactions [51]. Many protein domains often have similar or identical folding patterns, even if they show great variations in their sequences.

As indicated in [52], multiple ice-binding domains may be responsible for the ability of the diverse AFPs to bind to ice crystals. Therefore, we perform the feature extraction work from the functional domain information through the following steps. First, the functional domain composition of each antifreeze protein in the training dataset is obtained from the Intepro database [53]. Then, functional domains present in more than or equal to 10 antifreeze proteins are chosen to extract features. The result covers a total of 15 Intepro entries, as listed in Table 7, which may contribute to the AFP-ice interactions. Finally, the information of each of the 15 functional domains is represented by a binary score: 1 if present and 0 otherwise.

**Table 7:** The 15 Intepro entries that are present in more than or equal to 10 antifreeze proteins in the training dataset.

Rank	Intepro Entries	Rank	Intepro Entries	Rank	Intepro Entries
1	IPR001304	6	IPR000742	11	IPR000152
2	IPR016186	7	IPR000436	12	IPR001881
3	IPR016187	8	IPR000538	13	IPR003599
4	IPR018378	9	IPR007110	14	IPR018097
5	IPR013032	10	IPR013783	15	IPR013106

#### 3.2.5. Evolutionary Information

Evolutionary conservation is one of the most important aspects in biological sequence analysis. A more conserved residue prefers to locating at a functionally important region [54]. Protein evolution involves changes of single residues, insertions and deletions of several residues, gene doubling and gene fusion. With these changes accumulated for a long period of time, many similarities between initial and resultant protein sequences are gradually eliminated, but the corresponding proteins may still share many common features [26]. Protein sequences’ evolutionary conservation serves as evidence for structural and functional conservation [55]. To incorporate the evolutionary information of proteins, the position-specific scoring matrix (PSSM) [56] profiles are adopted here.

The PSSM is a matrix of score values generated from PSI-BLAST with 3 iterations and a cutoff *E*-value of 0.001. The rows and columns of the generated PSSM matrix are indexed by the protein residues and the 20 native amino acids, respectively. PSSM can be expressed for a protein sequence *P* with *L* residues as follows:

(4) $$P_{\mathrm{PSSM}}=\left[\begin{array}{cccccc} E_{1\to 1} & E_{1\to 2} & \cdots & E_{1\to j} & \cdots & E_{1\to 20}\\ E_{2\to 1} & E_{2\to 2} & \cdots & E_{2\to j} & \cdots & E_{2\to 20}\\ \vdots & \vdots & \cdots & \vdots & \cdots & \vdots \\ E_{i\to 1} & E_{i\to 2} & \cdots & E_{i\to j} & \cdots & E_{i\to 20}\\ \vdots & \vdots & \cdots & \vdots & \cdots & \vdots \\ E_{L\to 1} & E_{L\to 2} & \cdots & E_{L\to j} & \cdots & E_{L\to 20} \end{array}\right]$$

where $E_{i\to j}$ represents the score of the amino acid in the *i*-th position of the query sequence mutating to amino acid type *j* during the evolution process. Positive scores indicate that this change $E_{i\to j}$ occurs more frequently than expected occasionally, while negative scores indicate the opposite.

The elements of PSSM are scaled to the range from 0 to 1 using the following sigmoid function:

(5) $$f\left(x\right)=\frac{1}{1+e^{-x}}$$

where *x* is the original PSSM value.

We sum all of the rows and columns in the PSSM corresponding to the same functional group as given in Section 3.2.1 and then divide each element by the length of the sequence. In the prediction of AFPs, we use PSSM profiles to generate 100-dimensional (10×10 functional group pairs) input vectors as parameters.

### 3.3. Random Forest Classifier

The random forest (RF) algorithm, proposed by Breiman [57], has been successfully used in protein attribute prediction problems [58,59]. The RF is an ensemble classifier consisting of several decision trees. According to L. Breiman’s description [57], each tree is constructed through the following procedures. (i) Suppose the number of training cases is N; take N samples at random, but with replacement, from the original data. These samples are to form the training set for growing the tree; (ii) At every node of the tree, a feature subset with *m* features is randomly selected from all *n* features without replacement; (iii) Based on the randomly-selected samples and features, each tree is grown to the largest extent possible without pruning. To classify a new query sample, each decision tree yields a predicted class. The final classification of RF is obtained by combining the prediction results of all trees via voting.

WEKA (Waikato Environment for Knowledge Analysis), developed by the research team from University of Waikato in New Zealand, is free software integrating several state-of-the-art machine learning algorithms and data analysis tools [60]. In this study, the random forest classifier in WEKA software is employed to implement the classification and operated with the default parameters.

### 3.4. Ensemble Method

In classification problems, the composition of the training data has a significant effect on the classification accuracy. It is a remarkable fact that the data used to identify AFPs has an imbalanced class distribution, *i.e.*, the fraction of AFPs is relatively small compared to that of non-AFPs. The imbalanced data classification problem would result in high prediction accuracy for the majority class, but poor prediction accuracy for the minority class [61].

The existing methods for predicting AFPs [23,24,25] have tried to change the distribution of positive and negative samples by randomly selecting AFPs and non-AFPs with the same size as the training set. However, since only 300 non-AFPs were randomly selected from 9439 non-AFPs to form the negative samples of the training dataset, they failed to make full use of the negative sample information in the original dataset. This may lead to a biased estimate of the accuracy. Hence, it is urgent to adopt an effective method to get an unbiased prediction based on the imbalanced dataset.

Ensemble classification is a method to combine various basic classifiers that have independent decision-making abilities. Previous experimental results show that the ensemble method is often superior to the individual classifier, which enhances not only the performance of the classification, but also the confidence of the results [62,63]. In this paper, a random forest-based ensemble method is applied to address the imbalanced problem.

The prediction performance of the training dataset is evaluated by 10-fold cross-validation. During 10-fold cross-validation, samples in each class are partitioned into 10 none-overlapped subsets. Then, 9 subsets of each class are chosen as the training set, and the remaining one of each class as the testing set. The processes mentioned above are repeated 10 times. For each run, the whole procedures of the random sampling followed by the ensemble method are given in following steps.

In order to deal with this imbalanced data problem, the negative sample set is divided into multiple subsets to make sure that the number of samples of each subset is approximately equal to that of the positive samples. In addition, to reduce computational complexity, negative samples in the training set are randomly sampled to make sure that the sampled negative samples are *G* times the number of the positive samples in the training set. As the ratio of negative to sampled positive samples is *G*, the negative dataset in the training set is undersampled and split into *G* groups. Each group is then combined with the positive samples in the training set as a training subset. After the procedure, *G* training subsets are obtained. *G* random forest classifiers are trained by the *G* training subsets, respectively, and the performance of the model is evaluated by the testing set. The final predicted class is determined by majority voting among the outputs of the *G* classifiers. In the majority voting scheme, a test instance is labeled the predicted class that obtains the highest number of votes.

### 3.5. Feature Selection

After carrying out the above-mentioned feature extraction methods, all protein sequences are converted into numerical feature vectors with the same dimension. As we know, not all of the extracted features could contribute to the classification. Generally, the high dimensional vector in a feature set would cause 3 problems: over-fitting, information redundancy and dimension disaster [64]. Therefore, it is necessary to select high discriminative features, to reduce noise and, consequently, enhance the speed and performance with feature selection techniques. In this study, the analysis of variance followed by incremental feature selection (ANOVA-IFS) method is performed to pick out informative features from the original feature set.

The analysis of variance (ANOVA), proposed by Fisher [65], is a statistical technique to investigate the relationship between a response variable and one or more independent variables. The ANOVA is able to identify factors that statistically contribute to the dataset’s variability. Based on the ANOVA, a statistical test called the F test is usually used to measure the relevance of a feature with respect to groups. The *F* value of the τ-th feature in the feature set is defined as:

(6) $$F\left(\tau \right)=\frac{MS_{B}\left(\tau \right)}{MS_{W}\left(\tau \right)}$$

where $MS_{B}\left(\tau \right)$ is the τ-th feature variance between groups and $MS_{W}\left(\tau \right)$ is the τ-th feature variance within groups. They are expressed as:

(7) MSBτ=SSBτSSBτdfBdfB,MSWτ=SSWτSSWτdfWdfW

where $df_{B}=k-1$ and $df_{W}=N-k$ are the degrees of freedom for $MS_{B}$ and $MS_{W}$, respectively. *k* and *N* represent the number of groups and the total number of observations, respectively. $SS_{B}\left(\tau \right)$ and $SS_{W}\left(\tau \right)$ are the sums of squares of the τ-th feature between groups and within groups, respectively, and are calculated by:

(8) $$\begin{array}{c} SS_{B}\left(\tau \right)=\sum_{i=1}^{k}n_{i}{\left({\bar{y}}_{i}\left(\tau \right)-\bar{y}\left(\tau \right)\right)}^{2},\\ SS_{W}\left(\tau \right)=\sum_{i=1}^{k}\sum_{j=1}^{n_{i}}{\left(y_{ij}\left(\tau \right)-{\bar{y}}_{i}\left(\tau \right)\right)}^{2} \end{array}$$

where ${\bar{y}}_{i}\left(\tau \right)$ denotes the mean value of the τ-th feature in the *i*-th group, $\bar{y}\left(\tau \right)$ denotes the mean value of all of the observations of the τ-th feature, $y_{ij}\left(\tau \right)$ is the *j*-th observation of the τ-th feature in the *i*-th group, and $n_{i}$ denotes the number of observations in the *i*-th group.

The *F* value will become large as the $MS_{B}$ becomes increasingly larger than the $MS_{W}$. The feature with a larger *F* value indicates that it is a more highly relevant one for the target to be predicted. In other words, predicted groups have a stronger correlation with the $\tau_{1}$-th feature than with the $\tau_{2}$-th feature if $F\left(\tau_{1}\right)>F\left(\tau_{2}\right)$. The features used in this study then can be ranked by the *F* value.

Based on the ranked feature list evaluated by the ANOVA approach, the IFS method is adopted to determine the optimal feature set. The IFS procedure [66] starts with an empty feature set and adds features one by one from higher to lower rank. A new feature set is constructed when another feature has been added. Suppose the total number of the features is *N*, then we can obtain *N* new feature sets. The *i*-th feature set is denoted as:

(9) $$S_{i}=\left\{f_{1},f_{2},\cdots ,f_{i}\right\}\left(1\leq i\leq N\right)$$

For each of the *N* feature sets, an AFP-Ensemble-based predictor is constructed and examined using the 10-fold cross-validation on the training dataset. Thus, the optimal feature set can be obtained when the corresponding predictor yields the best performance.

### 3.6. Performance Measures

In statistical prediction, the following 3 cross-validation methods are often used to examine a predictor for its anticipated accuracy: independent dataset test, subsampling (K-fold cross-validation) test and jackknife test [67]. Among the 3 test methods, the jackknife test is deemed as the least arbitrary one that can always yield a unique result for a given benchmark dataset [29]. However, taking the size of the benchmark dataset into consideration, the 10-fold cross-validation test instead of the jackknife test is used in this study to reduce the computational time. Meanwhile, the independent dataset test is also adopted in our study.

Sensitivity (Sn), specificity (Sp), accuracy (Acc) and balanced accuracy (BAcc) are employed to evaluate the performance of the prediction system. These measurements are defined as follows.

(10) $$S_{n}=\frac{TP}{TP+FN}$$

(11) $$S_{p}=\frac{TN}{TN+FP}$$

(12) $$Acc=\frac{TP+TN}{TP+FP+TN+FN}$$

(13) $$BAcc=\frac{1}{2}\left(S_{n}+S_{p}\right)$$

where TP, FP, TN and FN represent true positive (correctly-predicted AFPs), false positive (non-AFPs incorrectly predicted as AFPs), true negative (correctly predicted non-AFPs) and false negative (AFPs incorrectly predicted as non-AFPs), respectively.

Sensitivity (Sn) measures the proportion of the known AFPs that are correctly predicted as AFPs, and specificity (Sp) measures the proportion of the known non-AFPs that are correctly predicted as non-AFPs. Accuracy (Acc) is the proportion of all samples that are correctly predicted. ACC is known to be inappropriate for an imbalanced dataset, since it may be high even if sensitivity or precision is low [68]. However, a good prediction system is usually expected to provide both high sensitivity and specificity. Therefore, the balanced accuracy (BAcc) is used throughout this study as the main evaluator for prediction performance.

## 4. Conclusions

The available evidence indicates that the antifreeze effect of overwintering organisms is largely due to a family of antifreeze proteins. The knowledge of antifreeze proteins is instructive for several industrial and biomedical applications. In this study, we develop a promising ensemble method called AFP-Ensemble to discriminate AFPs from non-AFPs by integrating a comprehensive set of feature descriptors. To obtain the optimal features, the ANOVA-IFS method is used for improving the prediction capability of the model. In view of the serious imbalance in the benchmark dataset, a random sampling approach followed by undersampling is adopted to rebuild multiple training subsets. Random forest classifiers are trained by different training subsets and then aggregated into a consensus classifier by majority voting. Experimental results show that AFP-Ensemble obtains satisfactory results. The sensitivity, specificity, accuracy and balanced accuracy are 0.892, 0.940, 0.938 and 0.916, respectively, for the independent testing dataset. Comparison results indicate that AFP-Ensemble performs far better than the previous studies, which suggests that AFP-Ensemble can serve as a useful tool to find the potential AFPs. The detailed feature analysis indicates that all feature types applied in this method contribute to the improved prediction performance. The findings derived from this paper may provide useful clues for further in-depth investigation into AFP-ice interactions and guide the related experimental validation.

## Supplementary Materials

Supplementary materials can be found at http://www.mdpi.com/1422-0067/16/09/21191/s1.
